# Antioxidant mediated protective effect of *Parthenium hysterophorus* against oxidative damage using *in vitro* models

**DOI:** 10.1186/1472-6882-13-120

**Published:** 2013-05-30

**Authors:** Shashank Kumar, Amita Mishra, Abhay K Pandey

**Affiliations:** 1 Department of Biochemistry, University of Allahabad, Allahabad, 211002, India

**Keywords:** Parthenium hysterophorus, Extracts, Phytochemicals, Phenolic, Reducing power, Hydroxyl radical scavenging activity, Lipid peroxidation

## Abstract

**Background:**

*Parthenium hysterophorus* L. (Asteraceae) is a common weed occurring throughout the globe. In traditional medicine its decoction has been used for treatment of many infectious and degenerative diseases. This work was therefore designed to assess the phytochemical constitution of *P. hysterophorus* flower and root extracts and to evaluate their reducing power, radical scavenging activity as well as protective efficacy against membrane lipid damage.

**Methods:**

Dried flower and root samples were sequentially extracted with non-polar and polar solvents using Soxhlet apparatus. The phytochemical screening was done using standard chemical methods and thin layer chromatography. Total phenolic content was determined spectrophotometrically. Reducing power and hydroxyl radical scavenging activity assays were used to measure antioxidant activity. Protection against membrane damage was evaluated by inhibition of lipid peroxidation (TBARS assay) in rat kidney homogenate.

**Results:**

Flavonoids, terpenoids, alkaloids and cardiac glycosides were present in all the extract. The total phenol contents in flower and root extracts were found to be in the range 86.69-320.17 mg propyl gallate equivalent (PGE)/g and 55.47-253.84 mg PGE/g, respectively. Comparatively better reducing power was observed in hexane fractions of flower (0.405) and root (0.282). Benzene extract of flower and ethyl acetate fraction of root accounted for appreciable hydroxyl radical scavenging activity (75-77%). Maximum protection against membrane lipid peroxidative damage among flower and root extracts was provided by ethanol (55.26%) and ethyl acetate (48.95%) fractions, respectively. Total phenolic content showed positive correlations with reducing power and lipid peroxidation inhibition (LPOI) % in floral extracts as well as with hydroxyl radical scavenging activity and LPOI % in root extracts.

**Conclusion:**

Study established that phytochemicals present in *P. hysterophorus* extracts have considerable antioxidant potential as well as lipo-protective activity against membrane damage.

## Background

Phytochemicals function as antioxidants and react with free radicals to combat oxidative stress. Excessive free radicals are generated in body due to unbalanced oxidants and antioxidants ratio which results into oxidative stress. Antioxidants scavenge and control the formation of free radicals thereby preventing oxidative damage to cellular components arising as a consequence of chemical reactions involving reactive oxygen species (ROS) [1,2]. Most of the antioxidants isolated from higher plants are polyphenols [3,4]. Evaluation of polyphenols and antioxidant activity of herbal products has become important tool to understand the medicinal property of plants. The antioxidant activity of phenolics is mainly due to their redox properties, which allow them to act as reducing agents, hydrogen donors, and singlet oxygen quenchers. In addition, they have a metal chelation potential. Polyphenols have carbon-based aromatic phenyl-ring compounds which are easily oxidized to quinones by ROS, a property that helps account for their free radical scavenging capacity. Phenolic compounds also possess potentially beneficial lipoxygenase inhibitory property [5].

In recent years study of lipid per-oxidation (LPO) is attracting much attention due to its role in disease processes. Having polyunsaturated fatty acids, membrane lipids are more likely prone to LPO. The membrane damage disrupts the functioning of the cell and its survival thus maintenance of its integrity is of greater importance. Lipid per-oxidation has been implicated in the pathogenesis of a number of diseases [6]. Aldehyde and other by-products of LPO are involved in physiological and pathological conditions of liver, kidney and brain toxicity [7,8]. Several other toxic by-products of LPO damage biomolecules away from the site of generation [9]. Removal of excess reactive species, suppression of their generation or protection against per-oxidation by repair of membrane damage may be an efficient way of preventing diseases. Agents that can interact with these secondary radicals formed during per-oxidation and scavenging them, would be effective in inhibiting LPO and in turn protect against free radical induced damage. Recent research has confirmed that antioxidants are the most effective tools to eliminate free radicals which cause oxidative stress and are possible protective agents that protect the cells from ROS and retard the progress of many diseases as well as lipid peroxidation [10].

*Parthenium hysterophorus* L. (Asteraceae), also known as congress grass, is an invasive weed throughout India and world. It is an aggressive colonizer of wastelands, pastures and roadsides in India. It suppresses yield of agricultural crops up to 50%-55% (>5-10 million rupees per annum) by competing strongly with their growth as well as contaminating the grain samples and a 90%-92% reduction in forage production (1–2 million rupees per annum) in India [11]. All parts of the plant are reported to be used as bitter tonic, febrifuge, emmenagogue, antidysenteric etc. [12]. In Maharashtra and Gujarat (India) the plant is used in the treatment of diabetes mellitus [13]. The plant has also been reported to have antibacterial [14] and anti tumor activity [15]. Therefore, the aim of present study was to evaluate chemical composition, assessment of antioxidant and bio-membrane protective activities of *P. hysterophorus* flower and root extracts.

## Methods

### Chemicals

Ethylene diamine tetra acetic acid (EDTA), ascorbic acid, trichloroacetic acid (TCA), dimethyl sulfoxide (DMSO), ferrozine, ferrous ammonium sulfate, propyl gallate, butylated hydroxyl anisole (BHA), butylated hydroxy toluene (BHT) were purchased from Himedia Laboratories Pvt. Ltd. (India). Folin-Ciocalteau reagent (FCR), potassium ferricyanide, silica gel-G were procured from Merck India. The remaining chemicals and solvents used were of standard analytical grade.

### Plant material and preparation of extracts

*P. hysterophorus* flower (PHF) and root (PHR) samples were collected during May 2010 from Science Faculty Campus, University of Allahabad, Allahabad, India. Identification of the plant was confirmed by Prof. D. K. Chauhan, Department of Botany, University of Allahabad and a voucher specimen has been deposited in the departmental herbarium (AU/BCH/AKP/07). The shade-dried plant samples were crushed and ground into fine powder using mortar and pestle. Powdered samples were sequentially extracted with hexane (HX), benzene (BZ), chloroform (CH), ethyl acetate (EA), acetone (AC), ethyl alcohol (ET) and water (AQ) in a Soxhlet apparatus for 6-8h [16]. The extracts were centrifuged and filtered. Solvent was removed completely under reduced pressure and dried residues were dissolved in DMSO or in respective solvents for determination of biochemical activities.

### Phytochemical screening

Various phytoconstituents viz., tannins, flavonoids, terpenoids, cardiac glycosides, anthraquinones, reducing sugars, alkaloids, phlobatannins and saponins present in PHF and PHR fractions were identified by using standard protocols [17-19].

### Thin layer chromatography (TLC)

TLC plates coated with silica gel G were prepared, dried and activated at 110°C for 90 min followed by cooling. The extracts were dissolved in respective solvents and spots were applied with the help of fine capillary tubes. A mixture of chloroform-ethyl acetate-formic acid (163:63:25) was used as the solvent system after several trials. The phytoconstituents were visualized as bands on chromatogram and retardation factor (R_F_ value) was calculated. The chromatogram was developed with spraying of FCR (1:1 in water) to identify the bluish bands having phenolic contents.

### Preparation of kidney homogenate

The kidney was isolated from normal albino Wistar rats and 10% (w/v) homogenate was prepared in phosphate buffer (0.1 M, pH 7.4 having 0.15 M KCl) using homogenizer (REMI motors Ltd., India) at 4°C. The homogenate was centrifuged at 800 g for 15 min and clear cell-free supernatant was used for the study of *in vitro* lipid per-oxidation (LPO) as an indicator of membrane damage.

### Determination of total phenolics

Total phenolic content in extract fractions was determined according to the protocol [20,21] with some modifications [16]. Modification included dissolution of extracts in DMSO instead of water. 0.2 ml of sample (2mg/ml in DMSO) was diluted to 3 ml with water. Small amount (0.5 ml) of two-fold-diluted FCR was added and the contents were mixed. After 3 min, 2 ml of 20% sodium carbonate solution was added and the tubes were placed in boiling water bath for one min followed by cooling. The absorbance was measured at 650 nm against a reagent blank using spectrophotometer (Visiscan 167, Systronics). The concentration of phenols in the test samples was expressed as mg propyl gallate equivalents/g sample (mg PGE/g). The estimation was performed in triplicate, and the results were expressed as mean ± SD.

### Hydroxy radical scavenging activity (HRSA)

HRSA of extracts was determined by the method of Klein et al. [22]. Aliquots of extracts (100 μl) were taken in different test tubes. One milliliter of Fe–EDTA solution (0.13% ferrous ammonium sulfate and 0.26% EDTA), 0.5 ml of 0.018% EDTA and 1 ml of 0.85% (v/v) DMSO (in 0.1M phosphate buffer, pH 7.4) were added to the test tubes, followed by 0.5 ml of 0.22% (w/v) ascorbic acid. The tubes were capped tightly and incubated on a water bath at 85°C for 15 min. After incubation test tubes were uncapped and 1 ml of ice-cold TCA (17.5% w/v) was added immediately. Three milliliters of Nash reagent (7.5 g of ammonium acetate, 3 ml glacial acetic acid and 2 ml acetyl acetone were mixed and made up to 100 ml with distilled water) was added to all the tubes and incubated further at room temperature for 15 min. Absorbance was measured at 412 nm. BHT was used as standard compound for comparison. Percentage (%) HRSA was calculated by the following formula:

$$\%\;HRSA\;=\left[A_{0}-A_{1}/A_{0}\right]\times 100$$

Where A_0_ is absorbance of the control and A_1_ is that of the individual samples or standard (s).

### Reducing power assay

The reducing power of test extracts was determined by the methods of [23,24] with slight modifications [25]. One ml aliquots of extracts (200–1000 μg/ml) prepared in DMSO was taken in test tubes. To each test tube 2.5 ml of phosphate buffer (0.2 M, pH 6.6) and 2.5 ml of 1% potassium hexacyanoferrate (K_3_Fe (CN)_6_) were added and contents were mixed. Tubes were incubated at 50°C in a water bath for 20 min. The reaction was stopped by adding 2.5 ml of 10% TCA and then centrifuged at 4000 g for 10 min. One ml of the supernatant was mixed with 1 ml of distilled water and 0.5 ml of FeCl_3_ solution (0.1%, w/v) and kept at 25°C for 2 min. The absorbance was measured at 700 nm. The ascorbic acid was used as positive control. All the tests were run in triplicate and results were reported as mean± SD.

### TBARS assay (Lipid per-oxidation Inhibition assay)

The lipo-protective efficacy of extracts in albino Wistar rat kidney homogenate was estimated by the method of [26] using some modification [27]. 100 μl extracts (2 mg/ml) dissolved in respective solvents were taken in test tubes and evaporated to dryness followed by addition of 1 ml KCl (0.15M) and 0.5 ml of kidney homogenate. Per-oxidation was initiated by adding 100 μl FeCl_3_ (0.2 mM). After incubation at 37°C for 30 min, lipid peroxidation was monitored by the formation of thiobarbituric acid reactive substances (TBARS). TBARS were estimated by adding 2 ml of ice-cold hydrochloric acid (0.25 N) containing 15% TCA, 0.38% TBA and 0.5% BHT. The reaction mixture was incubated at 80°C for 1 h then cooled and centrifuged. The absorbance of the pink supernatant (malondialdehyde formed by the lipid per-oxidation product and TBA complex) was measured at 532 nm. BHA was used as standard for comparison. All analyses were carried out in triplicate and results were expressed as mean ± SD. The protective effect of different extracts against lipid peroxidation (% LPOI) was calculated by using the following formula:

$$\mathrm{LPOI}\left(\%\right)=\left[1-A_{S}/A_{C}\right]\times 100$$

Where *A*_C_ is the absorbance of control and *A*_S_ is the absorbance of the standards or samples.

### Statistical analysis

All the experiments were performed in triplicate and results were expressed as mean ± SD. The graphs were plotted using Graph pad prism software. Linear regression analysis was performed to quote correlation coefficients between different parameters using Microsoft office excel 2007.

## Results

### Phytochemical analysis

Results of phytochemical screening are shown in Table 1. All the extracts tested positive for flavonoids, terpenoids and alkaloids. HX fractions of flower and roots were devoid of cardiac glycosides. Anthraquinone was present in all PHR fractions while among PHF extracts it was present in BZ, CH and AQ fractions only. Phlobatannins and saponins showed similar pattern of distribution among root extracts. Other phytochemicals did not produce uniform distribution.

**Table 1:** **Phytochemical investigation of *P. hysterophorus *flower and root extracts**

**Phytochemicals**	**HX**	**BZ**	**CH**	**EA**	**AC**	**ET**	**AQ**
	**f /r**	**f /r**	**f /r**	**f /r**	**f /r**	**f /r**	**f /r**
Tannins	−/−	−/−	+/−	+/−	+/−	+/−	+/+
Flavonoids	+/+	+/+	+/+	+/+	+/+	+/+	+/+
Terpenoids	+/+	+/+	+/+	+/+	+/+	+/+	+/+
Cardiac glycosides	−/−	+/+	+/+	+/+	+/+	+/+	+/+
Anthraquinones	−/+	+/+	+/+	−/+	−/+	−/+	+/+
Reducing sugars	+/−	−/+	+/−	+/+	+/+	+/+	+/−
Alkaloids	+/+	+/+	+/+	+/+	+/+	+/+	+/+
Pholobatannins	+/−	−/+	−/−	+/+	−/+	−/−	+/+
Saponins	−/−	+/+	−/−	−/+	−/+	−/−	+/+

Abbreviations: *HX* hexane, *BZ* benzene, *CH* chloroform, *EA* ethyl acetate, *AC* acetone, *ET* ethanol, *AQ* water. The signs (+ or -) before and after the slash indicate presence or absence of phytoconstituents in flower (f) and root (r) extracts, respectively.

### Thin layer chromatography

**Figure 1:**
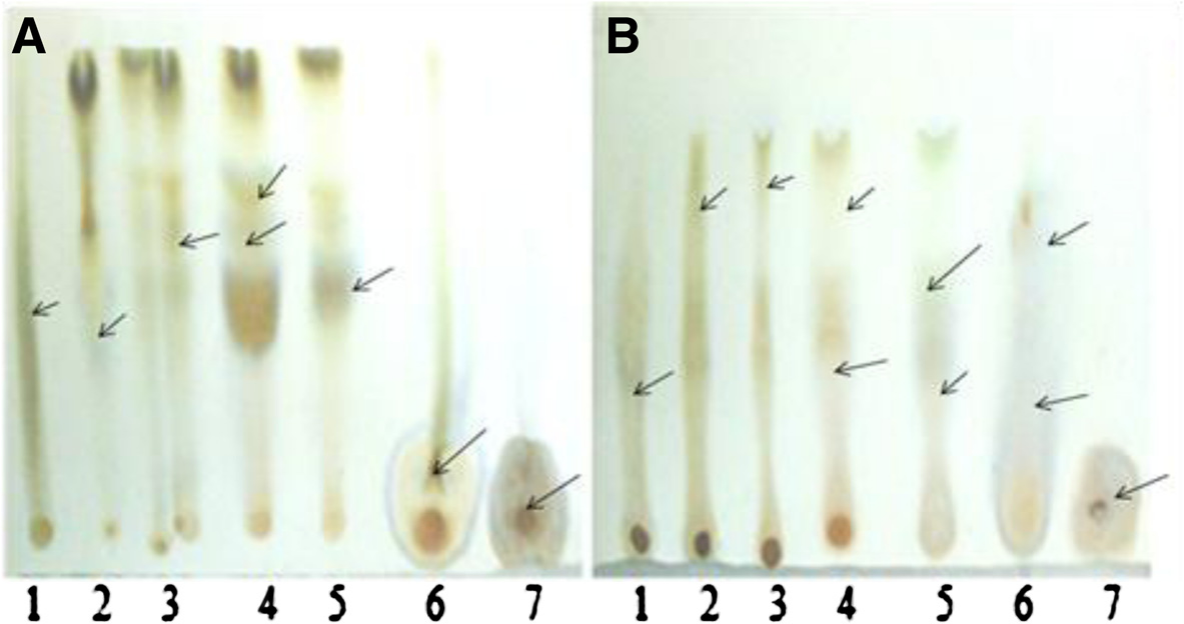
**Thin layer chromatogram of *P*. *hysterophorus *extracts.** (**A**) Flower and (**B**) Root extracts. The extracts dissolved in respective solvents were chromatographed in solvent system containing chloroform (163): ethyl acetate (63): formic acid (25) followed by spraying of FCR for visualization of phenolic contents. Arrows indicate phenolic spots. Numbers indicate specific extracts (1-hexane, 2-benzene, 3-chloroform, 4-ethyl acetate, 5-acetone, 6-ethyl alcohol, 7-aqueous extracts).

Several bands were observed during partitioning of extract components with solvent system (chloroform:ethylacetate:formic acid) indicating separation of phytoconstituents depending on polarity. After spraying FCR, phenolics were identified as bluish bands (Figure 1) on chromatogram. PHF extracts accounted for more diversity of phytochemicals than PHR extracts. R_F_ values of the bands are shown in Table 2.

**Table 2:** **Rf values of phenolic spots on thin layer chromatogram of *P. hysterophorus *flower and root extracts**

	**Bands**	**Extracts**						
		**HX**	**BZ**	**CH**	**EA**	**AC**	**ET**	**AQ**
Flower	1	0.43	0.40	0.60	0.63	0.58	0.28	0.00
	2	-	-		0.72		-	-
Root	1	0.41	0.93	0.99	0.45	0.41	0.41	0.00
	2	-	-	-	0.96	0.60	0.73	-

Abbreviations: *HX* hexane, *BZ* benzene, *CH* chloroform, *EA* ethyl acetate, *AC* acetone, *ET* ethanol, *AQ* water.

### Total phenolic content

Total phenolic contents in extracts are depicted in Table 3. Polar fractions in general accounted for appreciable phenolic contents. The phenolics ranged between 86–320 and 55–253 mg PGE/g, respectively in PHF and PHR extracts. The ET fractions of flower and root exhibited higher phenolic contents with values 320.17±0.65 and 253.84±0.41 mg PGE/g, respectively. The decreasing order of contents in PHF extracts was ET, AC, AQ, CH, BZ, EA and HX while in PHR extracts the order was ET, EA, AQ, CH, AC, BZ and HX, respectively.

**Table 3:** **Total phenolic content in *P. hysterophorus *flower and root extracts**

**Extracts**	**Flower**	**Root**
HX	86.69 ±0.88	55.47 ±0.26
BZ	119.24 ±0.41	71.14 ±0.51
CH	162.73 ±0.41	146.95 ±0.37
EA	107.22 ±0.40	230.27 ±0.26
AC	252.75 ±0.41	80.37 ±0.55
ET	320.17 ± 0.65	253.84 ±0.41
AQ	216.79 ±.058	159.21 ±0.34

Phenolic are represented as mg PGE/g of extract sample. Values are expressed as mean ± SD of three replicates. Abbreviations, *HX* hexane, *BZ* benzene, *CH* chloroform, *EA* ethyl acetate, *AC* acetone, *ET* ethanol, *AQ* water.

### Reducing power assay

**Figure 2:**
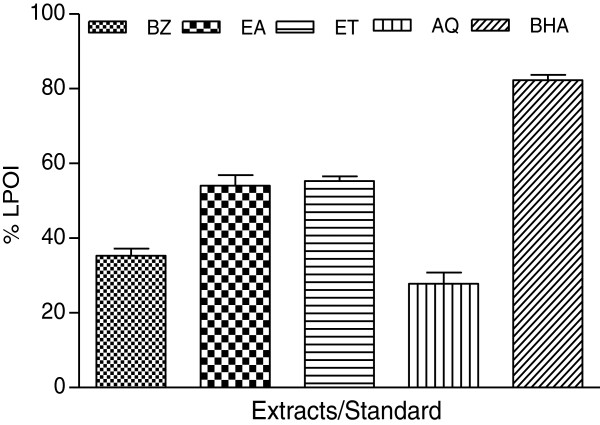
**Reducing power of *P. hysterophorus *flower extracts.** Extracts were prepared in hexane (HX), benzene (BZ), chloroform (CH), ethyl acetate (EA), acetone (AC), ethanol (ET) and water (AQ) as described in materials and methods section. Reducing power was measured at different concentration of extracts (200–1000 μg/ml). Butylated hydroxytoluene (BHT) was used as control. The results are expressed as mean ± SD of three replicates.

**Figure 3:**
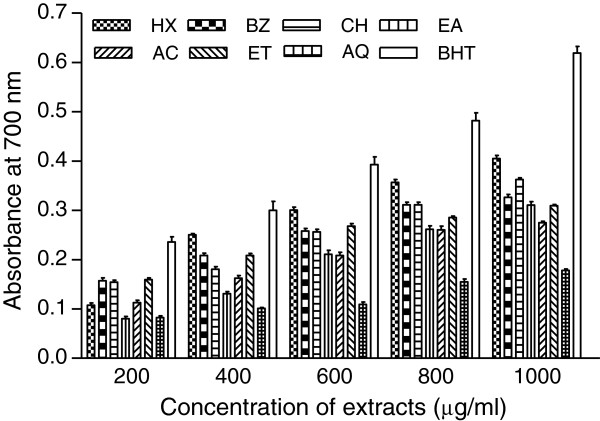
**Reducing power of *P. hysterophorus *root extracts.** Extracts were prepared in HX, BZ, CH, EA, AC, ET and AQ as described in materials and methods section. Reducing power was measured at different concentration of extracts (200–1000 μg/ml). BHT was used as control. The results are expressed as mean ± SD of three replicates.

Reducing potential of extracts was determined at different concentrations (200–1000 μg/ml). The results are shown in Figures 2 and 3, respectively. Comparatively better activity was observed in flower extracts. Reducing power increased with the increasing concentration of extracts. Among PHF fractions appreciable activity was found in HX followed by CH and other extracts. HX produced about 65% reducing power (0.405) as compared with the activity of BHT (0.619) while rest of the extracts accounted for about 50-59% activity at highest test concentration (Figure 2). However activities of HX and other extracts in comparison with standard compound at different concentrations (400–800 μg/ml) were found in the range 74-83% and 43-69%, respectively. Reducing power of PHR extracts at all test concentrations was lower than the activities of PHF extracts (Figure 3). The activity index of root extracts was found to be 37-61% with respect to the activity of BHT.

### Hydroxyl radical scavenging activity

Radical scavenging potential of flower and root extracts was determined in the concentration range 80–200 μg/ml and results are shown in Tables 4 and 5, respectively. Activities of most of the PHF extracts were comparable to the activities of BHT with few exceptions (CH and EA). An increasing trend in radical scavenging activity was observed with increasing concentration of all the extracts. The %HRSA values for most of the PHF extracts at a concentration of 200 μg/ml ranged between 70-78% except EA and CH (about 50%). Potential PHF extracts at all test concentrations demonstrated activity greater than 50% and exhibited dose dependent response (Table 4).

**Table 4:** **Hydroxyl radical scavenging activity (%) of *P. hysteroporus *flower extracts**

**Extracts**	**Concentration (μg/ml)**			
	**80**	**120**	**160**	**200**
HX	56.75 ±1.04	63.07 ±0.72	66.66 ±0.72	70.45 ±1.45
BZ	57.07 ±1.29	66.41 ±0.74	73.66 ±0.70	77.46 ±1.91
CH	34.57 ±1.01	38.41 ±1.15	43.81 ±2.32	55.04 ±1.25
EA	34.33 ±1.35	39.33 ±1.36	44.93 ±0.65	49.73 ±1.36
AC	45.51 ±0.69	54.53 ±1.48	68.44 ±1.28	73.57 ±1.59
ET	50.54 ±1.70	57.30 ±0.94	69.23 ±0.75	73.80 ±1.64
AQ	53.89 ±1.59	58.19 ±1.01	63.20 ±2.10	70.21 ±1.07
BHT	44.61 ±0.64	55.61 ±0.96	62.80 ±2.43	80.35 ±1.13

Values are expressed as mean ± SD of three replicates. Abbreviations, *HX* hexane, *BZ* benzene, *CH* chloroform, *EA* ethyl acetate, *AC* acetone, *ET* ethanol, *AQ* water, *BHT* Butylated hydroxyl toluene.

**Table 5:** **Hydroxyl radical scavenging activity (%) of *P. hysterophorus *root extracts**

**Extracts**	**Concentration (μg/ml)**			
	**80**	**120**	**160**	**200**
HX	25.66 ±1.03	30.55 ±1.02	46.41 ±0.80	63.52 ±0.76
BZ	0.82 ±1.23	25.47 ±1.89	43.03 ±1.32	56.15 ±1.07
CH	20.35 ±1.75	34.33 ±1.68	46.97 ±0.59	63.83 ±1.57
EA	47.84 ±1.24	58.52 ±0.96	64.07 ±1.23	74.79 ±1.31
AC	56.62 ±1.33	60.93 ±0.50	64.21 ±1.09	69.28 ±0.91
ET	56.16 ±0.95	60.87 ±0.75	63.47 ±1.66	68.97 ±0.44
AQ	61.99 ±1.28	65.01 ±0.54	68.44 ±0.54	71.57 ±1.26
BHT	44.61 ±0.64	55.61 ±0.96	62.80 ±2.43	80.35 ±1.13

Values are expressed as mean ± SD of three replicates. Abbreviations, *HX* hexane, *BZ* benzene, *CH* chloroform, *EA* ethyl acetate, *AC* acetone, *ET* ethanol, *AQ* water; *BHT* Butylated hydroxyl toluene.

Similar activity was also observed with PHR extracts. Appreciable activity was found in polar fractions at all the concentrations (Table 5). At 200 μg/ml, PHR extracts showed comparatively better activity viz., EA (74%), AC (69%), ET (68%) and AQ (71%). The observed activity of polar extracts at test concentrations was in the range 48-75%. Notable activity (43-64%) was also recorded in some of the non-polar fractions (BZ, HX and CH) at 160 and 200 μg/ml concentrations. Scavenging activity of PHF and PHR extracts were comparable to the activity shown by standard antioxidant BHT. The hydroxyl radical scavenging potential of BHT ranged between 44-80% at test concentrations.

### Lipid per-oxidation inhibition as a marker of bio-membrane protection

**Figure 4:**
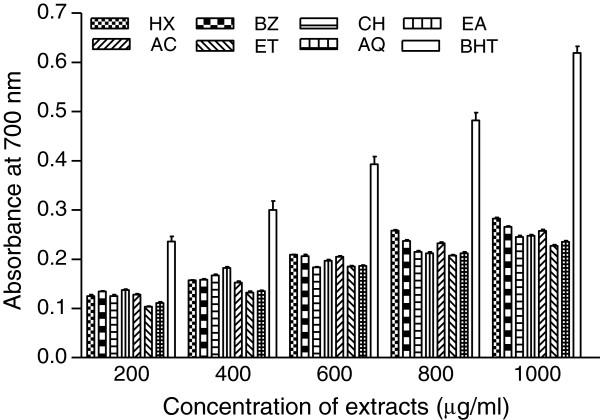
**Lipo-protective efficacy of *P. hysterophorus *flower extracts in tissue (rat kidney) homogenate.** Percent lipid per-oxidation inhibition (% LPOI) activity of flower extracts (BZ, EA, ET and AQ) at a concentration of 2 mg/ml was assessed as an indicator to protect per-oxidative damage of membrane lipids in rat kidney homogenate. Butylated hydroxylanisole (BHA) was used as control. The results are expressed as mean ± SD of three replicates.

**Figure 5:**
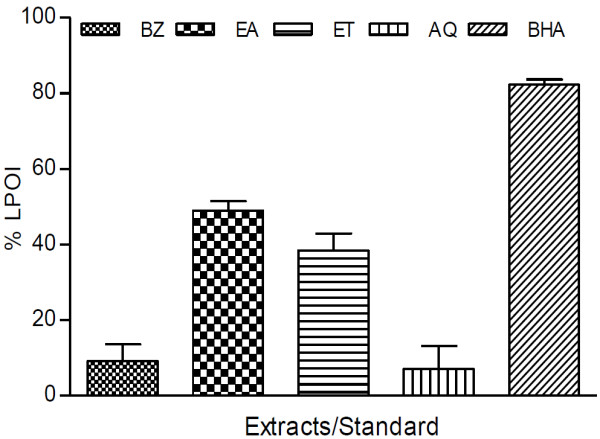
**Lipo-protective efficacy of *P. hysterophorus *root extracts in tissue (rat kidney) homogenate.** % LPOI activity of root extracts (BZ, EA, ET and AQ) at a concentration of 2 mg/ml was assessed as an indicator to protect per-oxidative damage of membrane lipids in rat kidney homogenate. BHA was used as control. The results are expressed as mean ± SD of three replicates.

Membrane protective efficacy of extracts in rat kidney tissue was assayed and % LPOI are shown in Figures 4 and 5. Among PHF extracts EA and ET fractions accounted for about 55% protection against membrane per-oxidative damage while %LPOI observed with other extracts was less than 36% (Figure 4). Similarly EA and ET fractions of PHR also showed comparatively better efficacy (Figure 5). The % LPOI for these fractions was about 49% and 38%, respectively while rest of the fractions could not provide protection against membrane damage. BHA showed better protective response (%LPOI about 80%).

## Discussion

Natural products continue to be one of the most important sources of lead compounds for the pharmaceutical industry. Plants have long been used as an alternative source for medicines and remedy for treating human diseases [28]. Present work describes the phytochemical characterization of various *P. hysterophorus* flower and root extracts, TLC profiling and antioxidant effects of extracts including membrane protective activity. Differences in the chemical constitution were observed in phytochemical screening of the test samples. Ubiquitous distribution of flavonoids, terpenoids, cardiac glycosides and alkaloids was found in PHF and PHR extracts. Rest of the phytoconstituents showed differential distribution pattern in polar and non-polar extracts.

Quantitative estimation of phenols in extracts demonstrated that PHF extracts possess comparatively higher amount of phenols (Table 3). Phenolic compounds are important phyto constituents and have potential against different diseases because of their antioxidant property [29]. They are known to possess antiviral, anti inflammatory, antiulcer, anti secretary, anti spasmodic, anti diarrheal and antitumor activities [30]. Radical scavenging via hydrogen atom donation by phenols is believed to be the predominant mechanism of antioxidant action. Other established mechanisms involve radical complexation of pro-oxidant metals as well as quenching through electron donation and singlet oxygen quenching. In our study it was observed that extracts possessing higher phenolic contents exhibited better biological activities. Several studies on total phenolic content had been published over the years demonstrating its importance in the medicinal field [27,31].

Flavonoids are a group of polyphenolic substances present in most plants and are responsible for various biochemical and antimicrobial activities [32]. Alkaloid content of the plants has been reported to produce antioxidant, anti-inflammatory and analgesic action [33]. Anthraquinones are wildly distributed in PHR extracts. Studies on other plants have also documented its higher concentration in roots [34,35]. Anthraquinones possess antioxidant [36] and anticancer properties [37]. Tannins and phlobatannins have been reported to have wound healing properties [38]. The biological importance of compounds such as saponins and cardiac glycosides has been well documented [39]. So the presence of various phytoconstituents in PHF and PHR extracts (Table 1) could be correlated with different types of biological activities.

Chromatogram developed with FCR showed several bluish bands indicating phenolic contents (Figure 1A and B). Many bands having similar Rf values (Table 2) were observed in different extracts of PHF and PHR suggesting presence of compounds having similar polarity.

Reducing power assay is based on the reduction of a Fe^3+^ complex to its Fe^2+^ form in the presence of antioxidants acting as electron donor [40,41]. Higher absorbance values indicated higher reducing power of extracts [25]. The experimental data (Figures 2 and 3) obtained in the present work showed marked reducing power in some of the extracts. Dose dependent activity of the extracts is further substantiated by the reports on other plants [42]. Total phenolic content in PHF and PHR extracts showed positive correlation with reducing power (Table 6). Thus the reducing activity of potential test extracts could be attributed to the presence of polyphenols [27], which may act in a similar fashion as reductones by donating the electrons and reacting with free radicals to convert them to more stable product [21].

**Table 6:** **Relationship among TPC, RP, %HRSA and %LPOI of *P. hysterophorus *flower and root extracts**

		**RP**	**%HRSA**	**% LPOI**
TPC	Flower	(+)/ r^2^ = 0.127	(−)/ r^2^ = 0.197	(+)/ r^2^ = 0.040
	Root	(−)/ r^2^ = 0.183	(+)/ r^2^ = 0.424	(+)/ r^2^ = 0.689
% LPOI	Flower	(+)/ r^2^ = 0.743	(+)/ r^2^ = 0.341	-
	Root	(+)/ r^2^ = 0.011	(+)/ r^2^ = 0.339	-
%HRSA	Flower	(+)/ r^2^ = 0.123	-	-
	Root	(−)/ r^2^ = 0.054	-	-

Signs in parentheses indicates positive (+) and negative (−) correlations. Abbrevations: *TPC* total phenolic content, *RP* reducing power, *HRSA* hydroxyl radical scavenging assay, *LPOI* lipid peroxidation inhibition, r^2^-regression coefficient.

In cell metabolism the hydroxyl radical (^**.**^OH) is the most reactive product of ROS formed by successive 1-electron reduction of molecular oxygen (O_2_). Hydroxyl radicals are primarily responsible for the cytotoxic effects in aerobic organisms [43]. These are generated in biological systems from H_2_O_2_ by the Fenton reaction. Results showed (Tables 4 and 5) that most of the fractions of PHF and PHR extracts have appreciable potential to scavenge the hydroxyl radical. Similar studies have been performed and reported on the protection from hydroxyl radical by different medicinal plants, thereby confirming antioxidant properties [44,45]. Hence, *P. hysterophorus* extracts can be used to minimize the adverse effects from the hydroxyl radicals. A negative correlation was observed for %HRSA with total phenolic content (r^2^=0.197) and positive correlation with reducing power (r^2^=0.123) of PHF fractions (Table 6). In PHR extracts positive correlation was observed between % HRSA and phenolic content (r^2^=0.424) while it was negative with reducing power (r^2^=0.054). Olabinri and his coworkers (2010) have also reported the negative correlation between the total phenolic content and %HRSA [46].

The short lived hydroxyl radicals are assumed to be more destructive to biomolecules because they are located less than a few nanometers from the site of generation [47]. Hydroxyl radicals have ability to interact with the purine and pyrimidine bases of DNA. It can abstract hydrogen atoms from biological molecules, including thiols, which leads into the formation of sulfur radicals capable to combine with oxygen to generate oxy-sulfur radicals [48]. Oxidative attack of hydroxyl radicals generated from Fenton reaction on deoxy-ribose produces malondialdehyde (MDA) and similar substances [49]. A characterized biologic damage by hydroxyl radical is its capacity to stimulate LPO, which occurs when OH radical is generated close to membranes and attacks the fatty acid side chains of the membrane phospholipids [48].

Lipid per-oxidation is a natural metabolic process under normal aerobic conditions and it is one of the most investigated consequences of ROS action on membrane structure and function. Polyunsaturated fatty acids (PUFA), the main components of membrane lipids, are susceptible to per-oxidation. Hydroxyl radicals and singlet oxygen can react with the methylene groups of PUFA forming conjugated dienes, lipid per-oxy radicals (LOOP) and hydro-peroxides (LOOH) [50]. Formation of conjugated dienes involves rearrangement of bonds [51]. The lipid hydro-peroxides produced can undergo reductive cleavage by reduced metals such as Fe^2+.^ and produces lipid alkoxyl radical which can initiate additional chain reactions [52]. The final stable products of per-oxidation are aldehydes which react with TBA to form thiobarbituric acid–malonaldehyde adduct with an absorbance maximum at 532 nm. In our studies presence of various *P. hysterophorus* extracts in LPOI *in vitro* model led to reduction of adduct formation indicating their lipo-protective potential. Other authors have revealed findings which are in agreement with our reports [53] while evaluating the protective effect of medicinal plants against membrane damage. This activity could be attributed to the hydroxyl radical scavenging by phytochemicals present in potent extracts. Moreover reducing power and chelation of metal ion could also be responsible for providing membrane protective efficacy in extracts.

Phenolics are a group of non-essential dietary components and their hydrogen donating property is responsible for the inhibition of free radical induced LPO [54,55]. A positive correlation was found between total phenolic content and % LPOI of PHF (r^2^= 0.040) as well as PHR (r^2^= 0.689) extracts. Similarly positive correlation was observed among %LPOI, reducing power and %HRSA of flower and root extracts (Table 6). The correlation index shows that phenolics are mainly responsible for producing lipo-protective activity in PHR extracts. In addition other non-phenolic phytochemicals present in extracts might also be involved in imparting some degree of membrane protection. Thus phenolic contents present in the PHF and PHR extracts could be accountable for their antioxidant and membrane protective activity.

## Conclusion

*P. hysterophorus* possess potential to neutralize the free radical induced oxidative damage. The work established considerable antioxidant and lipo-protective activity in various extracts. The study provides a step forward for further researches leading to development of antioxidant compounds of natural origin.

## Abbreviations

ROS: Reactive oxygen species; PHF: *Parthenium hysterophorus* flower; PHR: *P. hysterophorus* root; TBARS: Thiobarbituric acid reactive substances; PGE: Propyl gallate equivalents; LPOI: Lipid per-oxidation inhibition assay; HX: Hexane; BZ: Benzene; CH: Chloroform; EA: Ethyl acetate; AC: Acetone; ET: Ethyl alcohol; AQ: Water; FCR: Folin-Ciocalteau reagent; HRSA: Hydroxyl radical scavenging activity; MDA: Malondialdehyde.

## Competing interests

The authors declare that they have no competing interests.

## Authors’ contributions

AKP participated in the research design, analysis of the data and drafting of the manuscript. SK and AM conducted all the experiments and helped in drafting of manuscript. All authors have read and approved the final manuscript.

## Pre-publication history

The pre-publication history for this paper can be accessed here:

http://www.biomedcentral.com/1472-6882/13/120/prepub

## References

[B1] Percival M Anti-oxidants Clin Nutr Insight 1998 31 1 4

[B2] Pandey AK Mishra AK Mishra A Antifungal and antioxidative potential of oil and extracts derived from leaves of Indian spice plant *Cinnamomum tamala* Cell Mol Biol 2012 58 142 147 23273204

[B3] Larson RA The antioxidants of higher plants Phytochemistry 1988 27 969 978

[B4] Vinson JA Liang XQ Proch J Hontz BA Dancel J Sandone N Polyphenols antioxidants in citrus juices *in vitro* and *in vivo*studies relevant to heart diseases Adv Exp Med Biol 2002 50 5113 5122 10.1007/978-1-4757-5235-9_10 12083455

[B5] Sreejayan N Rao MNA Free radical scavenging activity of curcuminoids Arzneimittelforschung 1996 46 169 171 8720307

[B6] Lakshmi B Tilak JC Adhikari S Devasagayam TPA Janardhanan KK Inhibition of lipid peroxidation induced by *g*-radiation and AAPH in rat liver and brain mitochondria by mushrooms Current Science 2005 88 484 488

[B7] Devasagayam TPA Boloor KK Ramasarma T Methods for estimating lipid peroxidation: An analysis of merits and demerits (mini review) Ind J Biochem Biophys 2003 40 300 308 22900323

[B8] Nordberg J Arner ESJ Reactive oxygen species, antioxidants, and the mammalian thioredoxin system Free Rad Biol Med 2001 31 1287 1312 11728801 10.1016/s0891-5849(01)00724-9

[B9] Box HC Maccubbin AE Lipid peroxidation and DNA damage Nutrition 1997 13 920 921 9357035 10.1016/s0899-9007(97)00260-8

[B10] Kumar S Sharma UK Sharma AK Pandey AK Protective efficacy of *Solanum xanthocarpum* root extracts against free radical damage: phytochemical analysis and antioxidant effect Cell Mol Biol 2012 58 174 181 23273209

[B11] Knox J Jaggi D Paul MS Population dynamics of *Parthenium hysterophorus* (Asteraceae) and its biological suppression through *Cassia occidentalis* (Caesalpiniaceae) Turk J Bot 2011 35 111 119

[B12] Oudhia P Medicinal uses of Congress weed Parthenium hysterophorus L.: A Review 2001 Raipur, India: Society for Parthenium hysterophorus L Management (SOPAM) http://www.pankajoudhia.com/iprng/IPRNG-parthenium_a%26w12.htm

[B13] Patel VS Chitra V Prassanna PL Krishnaraju V Hypoglycemic effect of aqueous extract of *Parthenium hysterophorus* L. in normal and alloxan induced diabetic rats Ind J Pharmacol 2008 40 183 185 10.4103/0253-7613.43167 PMC2792614 20040954

[B14] Pandey AK Anti-staphylococcal activity of a pan-tropical aggressive and obnoxious weed *Parthenium histerophorus:* an in vitro study Natl Acad Sci Lett 2007 30 383 386

[B15] Reddy DM Qazi NA Sawant SD Bandey AH Srinivas J Shankar M Design and synthesis of spiro derivatives of parthenin as novel anti-cancer agents European J Med Chem 2011 46 3210 3217 21620534 10.1016/j.ejmech.2011.04.030

[B16] Mishra A Kumar S Bhargava A Sharma B Pandey AK Studies on *in vitro* antioxidant and antistaphylococcal activites of some important medicinal plants Cell Mol Biol 2011 57 16 25 21366958

[B17] Harborne JB Phytochemical Methods 1973 London: Chapman and Hall Ltd. 49 188

[B18] Mishra AK Misra A Kehri HK Sharma B Pandey AK Inhibitory activity of Indian spice plant *Cinnamomum zeylanicum* extracts against *Alternaria solani* and *Curvuluria lunata*, the pathogenic dematiaceous moulds Ann Clin Microbiol Antimicrobials 2009 8 9 10.1186/1476-0711-8-9 PMC2660280 19267932

[B19] Trease GE Evans WC Pharmacognosy 1989 11 London: Bailliere Tindall 45 50

[B20] Sadasivam S Manickam A Biochemical Methods 1996 2 New Delhi: New Age International (P) Ltd 193 194

[B21] Singh RP Chidambara Murthy KN, Jayaprakasha GK: Studies on the antioxidant activity of Pomegranate (*Punica granatum*) peel and seed extracts using *in vitro* models J Agric Food Chem 2002 50 81 86 11754547 10.1021/jf010865b

[B22] Klein SM Cohen G Cederbaum AI Production of formaldehyde during metabolism of dimethyl sulfoxide by hydroxyl radical generating systems Biochemistry 1981 20 6006 6012 6272833 10.1021/bi00524a013

[B23] Oyaizu M Studies on products of browning reactions: antioxidative activities of products of browning reaction prepared from glucosamine Japanese J Nutr 1986 44 307 315

[B24] Jayaprakasha GK Singh RP Sakariah KK Antioxidant activity of grape seed (*Vitis vinifera*) extracts on peroxidation models *in vitro* Food Chem 2001 73 285 290

[B25] Pandey AK Mishra AK Mishra A Kumar S Chandra A Therapeutic potential of *C. zeylanicum* extracts: an antifungal and antioxidant perspective Int J Biol Med Res 2010 1 228 233

[B26] Halliwell B Gutteridge JNC Halliwell B, Gutteridge JMC Mechanism of damage of cellular targets by oxidative stress: lipid peroxidation In Free Radicals in Biology and Medicine 1999 Oxford University Press UK: Oxford 284 313

[B27] Kumar S Pandey AK Antioxidant, lipo-protective and anti-bacterial activities of phytoconstituents present in *Solanum xanthocarpum* root Int Rev Biophysical Chem (IREBIC) 2012 3 42 47

[B28] Mishra AK Mishra A Bhargava A Pandey AK Antimicrobial activity of essential oils from the leaves of *Cinnamomum spp* Natl Acad Sci Lett 2008 31 341 345

[B29] Rice-Evans C Flavonoids antioxidants Curr Med Chem 2001 8 797 807 11375750 10.2174/0929867013373011

[B30] Carlo GD Mascolo N Izzo AA Capasso F Flavonoids: old and new aspects of a class of natural therapeutic drugs Life Sciences 1999 65 337 353 10421421 10.1016/s0024-3205(99)00120-4

[B31] Adeolu AA Florence OJ Anthony JA Patrick JM Antioxidant activities and phenolic contents of the methanol extracts of the stems of *Acokanthera oppositifolia* and *Adenia gummifera* BMC Comp Alt Med 2008 8 54 10.1186/1472-6882-8-54 PMC2566552 18817535

[B32] Miller AL Antioxidant flavonoids; structure, function and clinical usage Alt Med Rev 1996 1 103 111

[B33] Okwu DE Okwu ME Chemical composition of *Spondias mombin*L. plants parts J Sust Agric Environ 2004 6 140 147

[B34] Thomson RH Naturally occurring quinones 1971 London: Academic Press

[B35] Williams EM Major herbs of ayurveda 2002 Elsevier Science Ltd: Churchill Livingstone

[B36] Ammara RB Bhouri W Sghaier MB Boubaker J Skandrani I Neffati A Antioxidant and free radical-scavenging properties of three flavonoids isolated from the leaves of *Rhamnus alaternus* L. (Rhamnaceae): a structure– activity relationship study Food Chem 2009 116 258 264

[B37] Fenig E Nordenberg J Beery E Sulkes J Wasserman L Combined effect of aloe-emodin and chemotherapeutic agents on the proliferation of an adherent variant cell line of Merkel cell carcinoma Oncology Reports 2004 11 213 217 14654928

[B38] Kagbo HD Ejebe DE Phytochemistry and preliminary toxicity studies of the methanol extract of the stem bark of *Garcinia kola* (Heckel) Internet J Toxicol 2010 10 5580

[B39] Leverin G McMatron H Alkaloids and glycosides Clin Microbiol Rev 1999 11 156 250

[B40] Tachakittirungrod S Okonogi S Chowwanapoonpohn S Study on antioxidant activity of certain plants in Thailand: mechanism of antioxidant action of guava leaf extract Food Chem 2007 103 381 388

[B41] Yen GC Chen HY Antioxidant activity of various tea extracts in relation to their antimutagenicity J Agric Food Chem 1995 43 27 32

[B42] Jayasri MA Mathew L Radha A A report on the antioxidant activity of leaves & rhizome of Costus pietus D.Don Int J Integr Biol 2009 5 20 26

[B43] Halliwell B Gutteridge JMC Free Radicals in Biology and Medicine 1989 Oxford: Oxford University Press

[B44] Singh BN Singh BR Singh RL Prakash D Sarma BK Singh HB Antioxidant and anti-quorum sensing activities of green pod of *Acacia nilotica* L Food Chem Toxicol 2009 47 778 786 19168114 10.1016/j.fct.2009.01.009

[B45] Gul MZ Bhakshu LM Ahmad F Kondapi AK Qureshi IA Ghazi IA Evaluation of *Abelmoschus moschatus* extracts for antioxidant, free radical scavenging, antimicrobial and antiproliferative activities using *in vitro* assays BMC Comp Alt Med 2011 11 64 10.1186/1472-6882-11-64 PMC3201038 21849051

[B46] Olabinri BM Odedire OO Olaleye MT Adekunle AS Ehigie LO Olabinri PF In vitro evaluation of hydroxyl and nitric oxide radical scavenging activites of artemether Res J Biol Sci 2010 5 102 105

[B47] Hipeli S Elstner EF OH-radical-type reactive oxygen species: a short review on the mechanisms of OH-radical and peroxynitrite toxicity Z. Naturforsch C 1997 52 555 563 9373992

[B48] Halliwell B Reactive oxygen species in living systems: source, biochemistry and role in human disease Am J Med 1991 91 14S 22S 1928205 10.1016/0002-9343(91)90279-7

[B49] Devasagayam TPA Tilak JC Boloor KK Sane KS Ghaskadbi SS Lele RD Free radicals and antioxidants in human health: current status and future prospects JAPI 2004 52 794 804 15909857

[B50] Smirnoff N Smirnoff N Antioxidant systems and plant response to the environment Environment and plant metabolism: flexibility and acclimation 1995 BIOS Scientific Publishers: Oxford 217 243

[B51] Recknagel RO Glende EA Spectrophotometric detection of lipid conjugated dienes Methods Enzymol 1984 105 331 337 6727673 10.1016/s0076-6879(84)05043-6

[B52] Buettner GR The pecking order of free radicals and antioxidants: lipid peroxidation, α‒tocopherol, and ascorbate Arch Biochem Biophys 1993 300 535 543 8434935 10.1006/abbi.1993.1074

[B53] Khan RA Khan MR Ahmed M Sahreen S Shah NA Shah MS Bokhari J Rashid U Ahmed B Jan S Hepatoprotection with a chloroform extract of *Launaea procumbens* against CCl_4_-induced injuries in rats BMC Comp Alt Med 2012 12 114 10.1186/1472-6882-12-114 PMC3492157 22862950

[B54] Sheena N Ajith TA Janardhanan KK Anti inflammatory and antinociceptive activities of *Ganoderma lucidum* occurring in South India Pharmaceutical Biol 2003 41 301 304 10.1002/tcm.10065 12616600

[B55] Yen CC Duh PD The relationship between antioxidant activity and maturity of peanut hulls J Agric Food Chem 1993 41 67 70

